# Downregulation of intratumoral expression of miR-205, miR-200c and miR-125b in primary human cutaneous melanomas predicts shorter survival

**DOI:** 10.1038/s41598-018-35317-3

**Published:** 2018-11-20

**Authors:** Beatriz Sánchez-Sendra, Carolina Martinez-Ciarpaglini, José F. González-Muñoz, Amelia Murgui, Liria Terrádez, Carlos Monteagudo

**Affiliations:** 1 Department of Pathology, University of Valencia, Valencia, Spain; 2 Biomedical Research Institute INCLIVA, Valencia, Spain; 3 Department of Biochemistry and Molecular Biology, University of Valencia, Valencia, Spain; 4 Department of Pathology, Hospital Clínico Universitario de Valencia, Valencia, Spain

## Abstract

While only 15–25 percent of melanoma patients develop distant metastasis and die, this disease is still responsible for the majority of skin cancer-related deaths. The availability of adjuvant therapies makes the selection of high-risk patients essential. We evaluated the intratumoral expression of ten miRNAs in primary melanomas in relation to its ability to predict melanoma survival. To this end, we correlated miRNA expression in 132 cryopreserved primary and metastatic tumors with clinicopathological factors and clinical outcome. We found sequential downregulation of intratumoral expression of miR-125b, miR-182, miR-200c and miR-205 over the full spectrum of melanoma progression. Moreover, downregulation of these miRNAs occurred in primary melanomas that further disseminated to distant sites. Furthermore, miR-125b, miR-200c and miR-205 correlated as independent factors with shorter survival. Our *in vitro* findings demonstrate that loss of miR-205 potentiates the invasive ability of melanoma cells. We conclude that the downregulation of miR-205 in primary melanomas is an intrinsic property that might contribute to distant metastasis. In particular, the interaction of melanoma cells with the extracellular matrix is one of the key mechanisms by which miR-205 influences melanoma metastasis. In conclusion, miR-125b, miR-200c and miR-205 are useful prognostic biomarkers at the time of diagnosis to select high-risk patients.

## Introduction

Malignant melanoma already accounts for around 80% of all skin cancer-related deaths and its incidence is increasing worldwide^1^. Metastatic dissemination of cutaneous malignant melanoma is the main cause of the high mortality observed in melanoma.

There is cumulative evidence that miRNAs, which control many pathological processes acting as gene expression regulators, mediate melanoma invasion and metastasis. Here, we investigated the ability of a miRNA panel to select patients at high-risk of distant metastasis at the time of primary tumor diagnosis.

The current American Joint Committee on Cancer (AJCC) staging system for cutaneous melanoma is based on primary tumor thickness and the presence of ulceration, lymph node spread and distant metastases as determinants of prognosis^2^.

The identification of metastatic disease is a major factor for clinical outcome in patients with melanoma. While most melanoma patients never develop recurrent disease after excision of the primary tumor, a subset of patients (15–25%) will develop metastases and die from melanoma. However, clinical and histopathological features alone are currently unable to accurately predict clinical behavior in all melanoma cases.

Deregulation of some microRNAs and their targeted genes have been associated with the well-known hallmark biological characteristics of human cancer^3–6^. microRNAs (miRNAs) are endogenous small noncoding RNAs that regulate the stability or translational efficiency of target mRNAs and are involved in various biological processes, including differentiation, cell proliferation, apoptosis, transformation, invasion and migration of melanoma tumor cells^7,8^. miRNAs regulate the expression of more than 60% of protein coding genes and are aberrantly expressed in many human cancers contributing to the initiation and development of various types of cancer, including melanoma^9^. Depending on the cancer type they can function as tumor suppressor genes or oncogenes.

To date, most miRNA studies on melanoma have aimed to identify miRNAs capable of distinguishing normal tissue from melanoma tissue, or primary from metastatic disease, but few studies exist on miRNAs able to predict distant metastatic potential among primary melanomas. Our goal was to evaluate the ability to predict clinical outcome of the expression in the tumor microenvironment of human melanomas, of those miRNAs which have already been implicated in the literature in melanoma cell migration and/or invasion.

## Results

### miR-9, miR-125b, miR-137, miR-182, miR-200c and miR-205 expression correlate with prognostic clinicopathologic features in primary human melanoma

**Figure 1:**
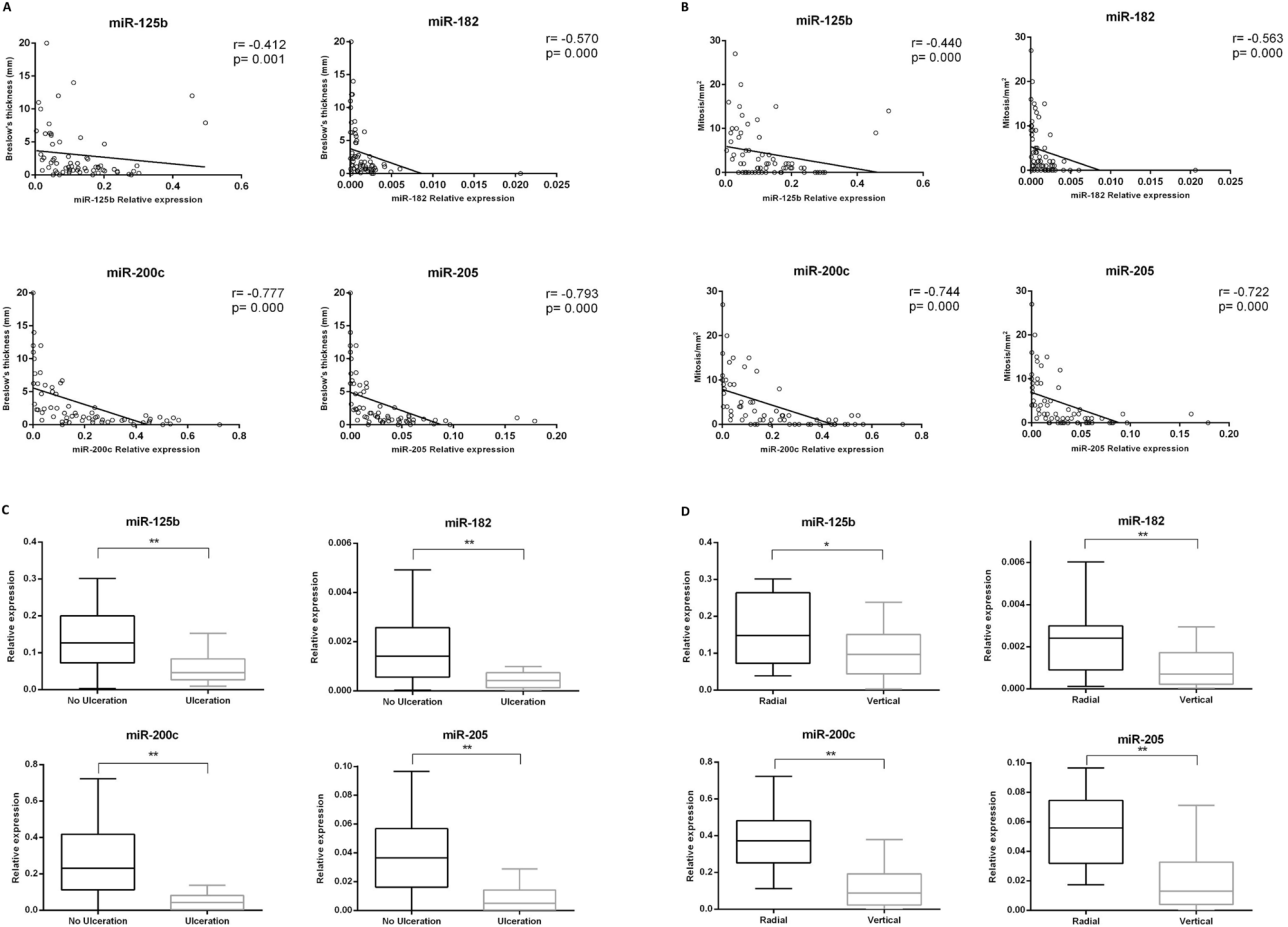
Correlation between miR-125b, miR-182, miR-200c and miR-205 expression and histopathologic features in primary melanomas. (**A**) Correlation of relative expression of miR-125b, miR-182, miR-200c and miR-205 according to Breslow thickness, (**B**) the number of mitoses per mm^2^, (**C**) ulceration (**D**) and growth phase. **p < 0.01 and *p < 0.05.

The clinicopathological features of the primary tumors included in our study are summarized in Table 1. We found a significant inverse correlation between miR-125b, miR-182, miR-200c and miR-205 expression and both Breslow thickness and mitotic index in primary melanomas (Spearman correlation) (Table 2). Similarly, ulcerated primary melanomas and those with vertical growth phase exhibited lower levels of miR-125b, miR-182, miR-200c and miR-205 (Mann-Whitney test) (Table 2) (Fig. 1A–D).

**Table 1:** Primary melanomas histological and clinical characteristics.

Primary melanomas (N = 65)		
Variable	Number of cases	(%)
Breslow thickness (mm)		
≤1	26	40.0
≥1	39	60.0
Ulceration		
Absent	48	73.8
Present	17	26.2
Mitosis/mm^2^		
0≥1	19	29.2
≥1	46	70.8
Growth phase		
Radial	20	30.8
Vertical	45	69.2
Location		
Limbs	24	36.9
Trunk	34	52.3
Head and neck	7	10.8
Gender		
Female	42	64.6
Male	23	35.4
Histological type*		
SSM	46	70.8
LMM	5	7.7
ALM	5	7.7
NM	9	13.8
Age at diagnosis (years)		
≤65	34	52.3
>65	31	47.7
Follow-up (months)		
Mean. Range	71	6.5–156.0

*Superficial Spreading Melanoma (SSM), Lentigo Maligna Melanoma (LMM), Acrolentiginous Melanoma (ALM) and Nodular Melanoma (NM).

**Table 2:** Relation between miRNA expression and clinicopathological markers.

Prognostic clinicopathological markers	Significant miRNAs	p value	Correlation coefficient	Test	Fold Change ^†^
Breslow thickness	**miR125b**	0.001**	**−0.412**	Spearman	0.498
	miR137	0.006**	0.336		1.269
	**miR182**	0.000**	**−0.570**		0.307
	**miR200c**	0.000**	**−0.777**		0.188
	**miR205**	0.000**	**−0.793**		0.175
	miR9	0.007**	0.329		1.805
Ulceration	**miR125b**	0.000**		Mann-Whitney	0.367
	miR137	0.007**			3.776
	**miR182**	0.001**			0.301
	**miR200c**	0.000**			0.188
	**miR205**	0.000**			0.141
	miR211	0.013*			0.406
	miR9	0.002**			3.215
Mitosis/mm^2^	**miR125b**	0.000**	**−0.440**	Spearman	0.391
	miR137	0.021*	0.287		1.751
	**miR182**	0.000**	**−0.563**		0.316
	**miR200c**	0.000**	**−0.744**		0.231
	**miR205**	0.000**	**−0.722**		0.192
	miR211	0.023*	−0.282		0.600
	miR9	0.007**	0.333		2.089
Growth phase	**miR125b**	0.030*		Mann-Whitney	0.653
	miR137	0.040*			1.575
	**miR182**	0.001**			0.294
	**miR200c**	0.000**			0.239
	**miR205**	0.000**			0.234
	miR9	0.014*			1.997
Location	**miR182**	0.027*		Kruskal-Wallis	1.893, 3.171
	**miR200c**	0.042*			2.294, 5.663
Gender	miR211	0.040*		Mann-Whitney	0.627
Histological type	**miR200c**	0.011*		Kruskal-Wallis	6.307, 9.477, 1.533
	**miR205**	0.012*			5.375, 8.286, 1.652

**Significant at p < 0.01. *Significant at p < 0.05. ^†^All miRNA expression ratios were calculated above/ below cut-off point. For continuous variables such as Breslow and number of mitosis, sample was split by median (Breslow median = 1.1 mm, mitosis median = 2). For categorical variables with two groups, fold change was calculated as presence versus absence of ulceration, vertical versus radial growth phase and males versus females. For location, a categorical variable with three groups, fold change is referred to the group with less expression for both miRNAs (limbs location), first fold change value is trunk/limbs and the second refers to head and neck/limbs. For histological type, a categorical variable with four groups, fold change refers to the group with less expression for both miRNAs (nodular melanomas), first fold change value is for superficial spreading melanomas, second for lentigo maligna and the third for acrolentiginous melanomas.

In contrast, cases with higher Breslow thickness, higher mitotic index, ulceration and vertical growth phase showed increased miR-9 and miR-137 expression (Table 2).

### Differential expression profile of miR-9, miR-125b, miR-137, miR-182, miR-200c and miR-205 in the different steps of melanoma progression

**Figure 2:**
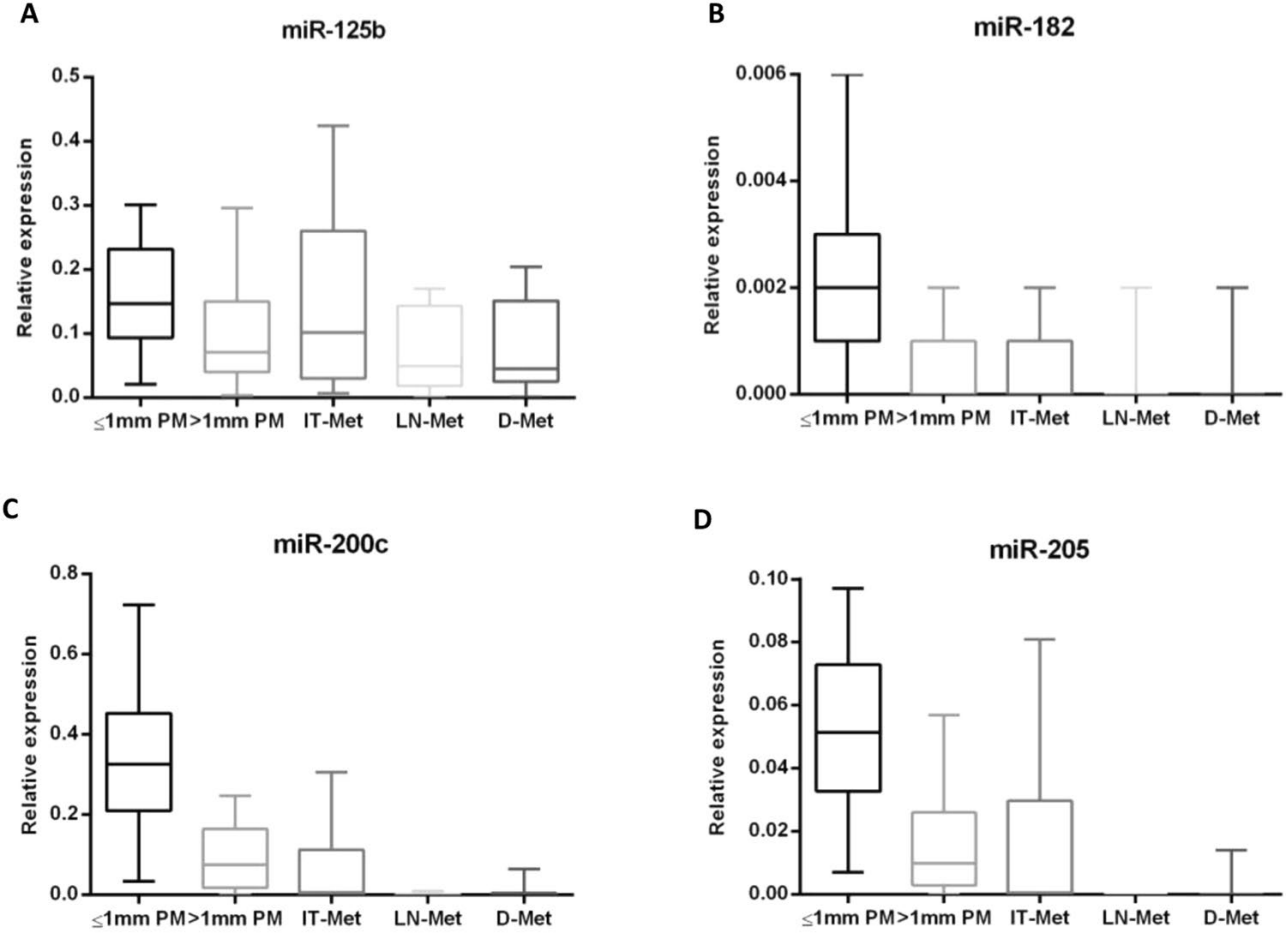
Comparison between the relative expression of miR-125b, miR-182, miR-200c and miR-205 along the different steps of melanoma progression. (**A**) Relative miR-125b, (**B**) miR-182, (**C**) miR-200c and (**D**) miR-205 expression in thin primary melanomas (≤1 mm), thick primary melanomas (>1 mm), in-transit metastases (IT-Met), lymph node metastases (LN-Met) and in distant metastases (D-Met).

Progressive loss of miR-125b, miR-182, miR-200c and miR-205 expression was found along the full spectrum of melanoma progression, from thin primary melanomas to distant metastatic tumors (miR-125b, p = 0.014; miR-182, p < 0.0001; miR-200c, p < 0.0001 and miR-205, p < 0.0001; Kruskal-Wallis test) (Fig. 2).

### Correlation of miR-125b, miR-182, miR-200c and miR-205 expression in primary tumors with further development of distant metastasis and melanoma specific survival

**Figure 3:**
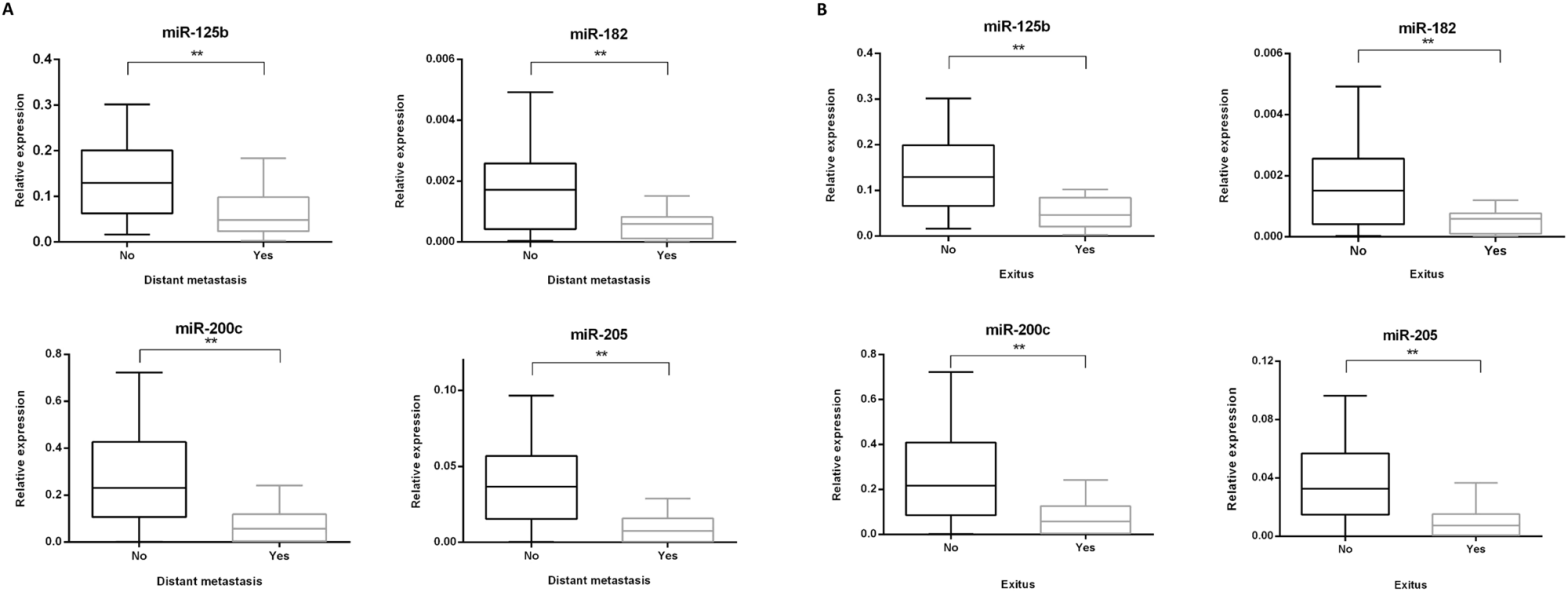
Association of miR-125b, miR-182, miR-200c and miR-205 expression in primary tumors according to clinical behavior. (**A**) Association of miR-125b, miR-182, miR-200c and miR-205 expression in primary tumors based on the further development of distant metastasis and (**B**) melanoma specific death. **p < 0.01.

Of the primary melanomas, 28% (18/65) further metastasized to distant sites, and 89% (16/18) of these patients died of melanoma (Table 3). miR-125b, miR-182, miR-200c and miR-205 expression was significantly lower in primary tumors which further metastasized to distant sites (p < 0.001, p < 0.01, p < 0.001 and p < 0.001, respectively) a tendency which was also found in primary tumors of the patients who died from melanoma (p < 0.001, p < 0.01, p < 0.001 and p < 0.001, respectively) (Fig. 3).

**Table 3:** Clinical progression of primary melanomas.

Primary melanomas (N = 65)		%	Day[Average (range)]
Variable	Number of cases		
In-transit Metastasis			**2,081.23 (0-4,759)**
Absent	56	86.2	
Present	9	13.8	
Lymph Node Metastasis			**2,029.18 (0-4,759)**
Absent	50	76.9	
Present	15	23.1	
Distant Metastasis			**2,118.8 (0-4,759)**
Absent	47	72.3	
Present	18	27.7	
Melanoma Specific Survival			**2,164.26 (197-4,759)**
Alive	49	75.4	
Dead	16	24.6

### Correlation of miR-125b, miR-182, miR-200c and miR-205 expression in primary tumors with distant metastasis free survival and melanoma specific survival

**Figure 4:**
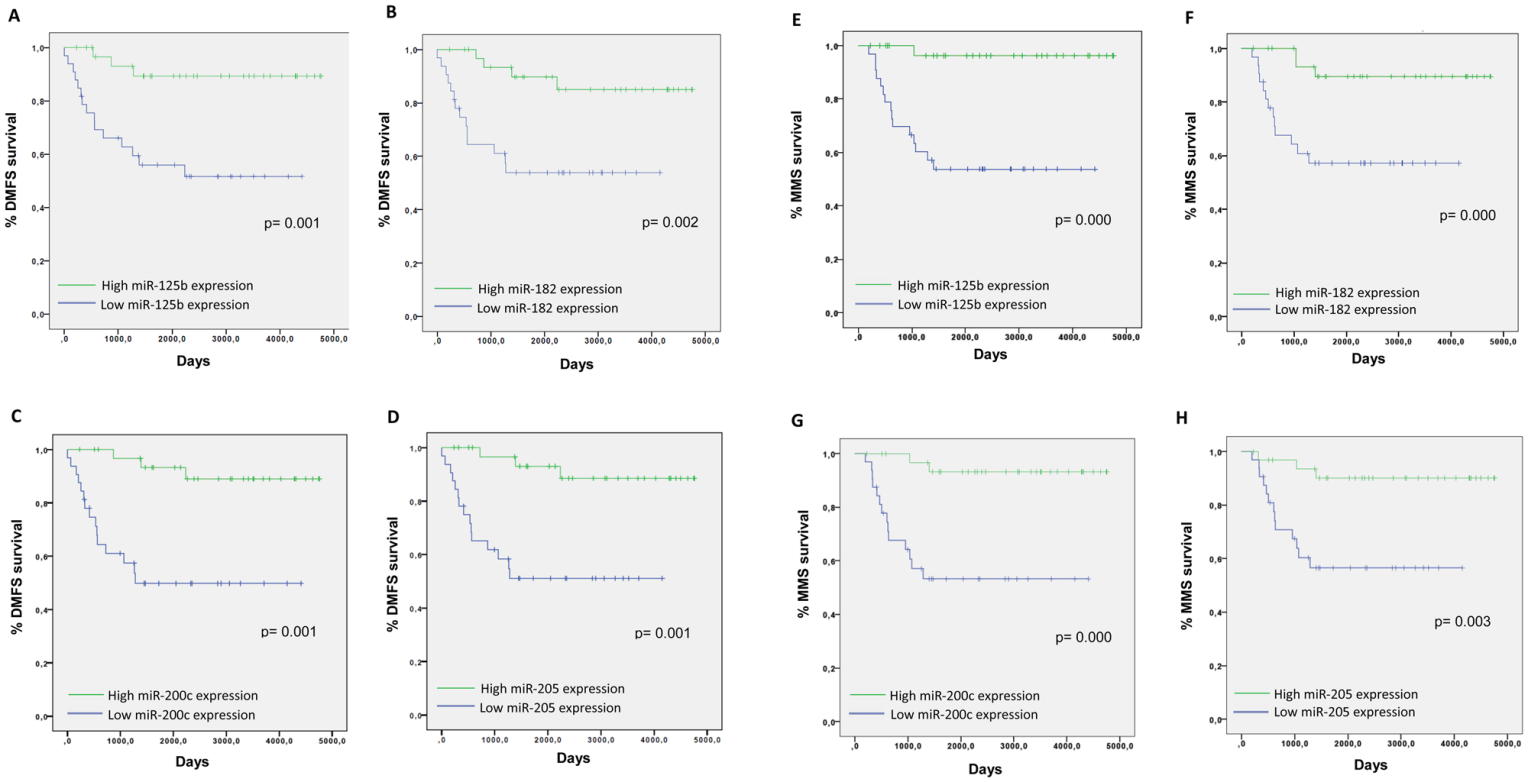
Kaplan-Meier curves for DMFS or MSS of melanoma patients. (**A–D**) Influence of miR-125b, miR-182, miR-200c and miR-205 expression affecting the DMFS of patients and (**E–H**) the influence of the same four miRNA expression on MSS.

In Kaplan-Meier survival analysis, both distant metastasis free survival (DMFS) and melanoma specific survival (MSS), were significantly longer in patients with primary melanomas showing miR-125b, miR-182, miR-200c and miR-205 expression levels above the median (Fig. 4, Table 4).

**Table 4:** Kaplan-Meier average survival times depending on miR-125b, miR-182, miR-200c and miR-205 expression being over or down the median expression values.

DMFS			MSS		
	Mean (days)	95% CI		Mean (days)	95% CI
**miR-125b**			**miR-125b**		
Down	2,611.03	1,940.25–3,281.83	Down	2,689.16	2,044.48–3,333.84
Over	4,344.70	3,900.75–4,788.66	Over	4,621.19	4,356.12–4,886.25
**miR-182**			**miR-182**		
Down	2,475.38	1,821.72–3,129.03	Down	2,637.76	2,009.08–3,266.43
Over	4,254.76	3,794.75–4,714.00	Over	4,382.28	3,978.51–4,786.05
**miR-200c**			**miR-200c**		
Down	2,467.11	1,762.25–3,171.97	Down	2,651.32	1,971.06–3,331.57
Over	4,405.86	4,026.23–4,785.48	Over	4,519.21	4,198.04–4,840.38
**miR-205**			**miR-205**		
Down	2,388.23	1,740.57–3,035.89	Down	2,638.33	2,012.78–3,263.87
Over	4,387.37	3,988.20–4,786.55	Over	4,379.73	3,970.61–4,788.85

Cox multivariate analysis was performed to elucidate if these four miRNAs were independent of the main clinicopathological prognostic variables. miR-125b, miR-200c and miR-205 expression were independent of Breslow, and miR-205 independent of Breslow and ulceration (Table 5). Overall, miR-205 was the best miRNA independent predictor of melanoma specific survival.

**Table 5:** Multivariate Cox regression analysis for MSS.

	HR (Exp(B))	95% CI	p value
Breslow			
Breslow	1.114	1.018–1.219	0.019*
miR-125b	0.987	0.976–0.998	0.018*
Breslow	1.106	1.008–1.214	0.033*
miR-182	0.510	0.247–1.051	0.068
Breslow	1.081	0.974–1.199	0.145
miR-200c	0.994	0.988–1.000	0.044*
Breslow	1.069	0.960–1.189	0.223
miR-205	0.955	0.916–0.996	0.032*
Ulceration			
Ulceration	5.109	1.689–15.456	0.004
miR-125b	0.991	0.982–1.000	0.062
Ulceration	5.562	1.873–16.511	0.002
miR-182	0.581	0.300–1.125	0.107
Ulceration	3.898	1.185–12.823	0.025
miR-200c	0.995	0.989–1.000	0.071
Ulceration	3.086	0.972–9.799	0.056
miR-205	0.953	0.914–0.994	0.027*

### miRNA expression in melanocytes and melanoma cell lines

Using RT-qPCR we tested the expression of the ten miRNAs in melanocytes and the following well-characterized melanoma cell lines: A375, SKMEL-147 and 451 Lu.

**Figure 5:**
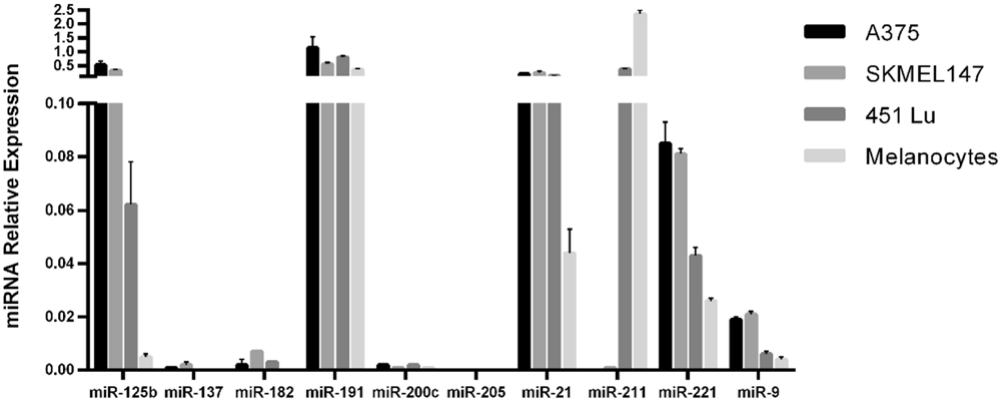
miRNA relative expression in melanoma cell lines and in melanocytes. Quantification of the ten selected miRNAs on A375, SKMEL147 and 451 Lu human melanoma cell lines and in melanocytes by reverse transcription quantitative Real time PCR analysis (RT-qPCR). All reactions were performed in triplicate and RNU48 was used as the reference control.

Our analysis showed that miR-205 (an independent prognostic factor for MSS) was the only miRNA which was not expressed in melanoma cell lines tested or melanocytes (Fig. 5). Compared with melanoma cell lines, melanocytes expressed lower miR-125b, miR-182 and miR-21 levels, did not express miR-137, and showed the highest miR-211 expression. miR-205 was therefore selected for *in vitro* functional assays in order to better describe the biological mechanisms affecting clinical outcome of melanoma patients.

### miR-205 overexpression in human melanoma cells

Our goal was to determine if miR-205 overexpression in human melanoma cells is associated with changes in the proliferation, migration and invasion ability of tumor cells *in vitro* after transduction.

**Figure 6:**
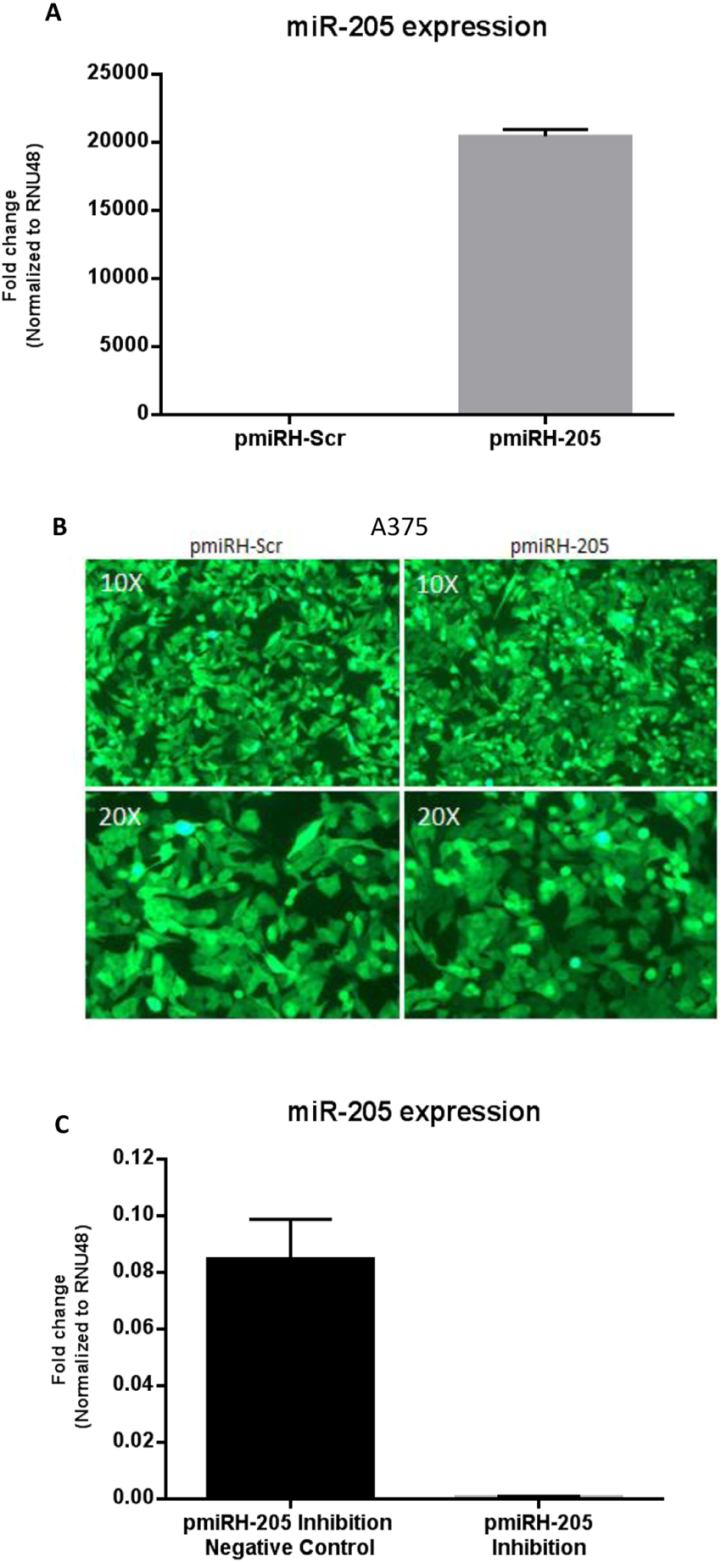
miR-205 overexpression and inhibition in A375 human melanoma cell line. (**A**) Quantitative RT‐qPCR validation of miR‐205 overexpression after transfection and later infection with pmiRH-205 expression vector or pmiRH-Scr (control) vector. Data is presented as mean ± SD (*n* = 3). (**B**) Fluorescent images of GFP positive cells showing strong GFP signal on the entire cells demonstrate an efficient transduction of the miR-205 precursor or the control vector. (**C**) Quantitative RT-PCR validation of miR-205 inhibition of A375 overexpressing miR-205.

Relative mature miR-205 expression levels in A375 cells after lentiviral transduction with pmiRH-205 or pmiRH-Scr constructs were determined by TaqMan miRNA RT-qPCR. Validation of miR-205 overexpression by RT-qPCR showed high levels of mature miR-205 in pmiRH-205 transduced cells and a lack of miR-205 expression in pmiRH-Scr cells. These experiments were performed in triplicate (Fig. 6A). A very close to 100% transduction efficiency was achieved as the vast majority of cells were fluorescent (Fig. 6B).

### miR-205 inhibition of human melanoma cells overexpressing miR-205

To exclude potential undesirable effects during the overexpression procedure we specifically inhibited miR-205 in cells overexpressing miR-205. miR-205 was inhibited approximately 16-fold upon inhibition which resulted to be significant (Fig. 6C).

### Functional validation of miR-205 overexpression and inhibition

We also evaluated the functional effect of miR-205 overexpression and inhibition at protein level by immunoblot densitometry after A375 cells transduction with pmiRH-205 or pmiRH-Scr and A375 overexpressing miR-205 transfected with mirVana miR-205 inhibitors or the mirVana inhibitor negative control. In melanoma, miR-205 represses expression of its direct target ZEB1. Thus, cells overexpressing miR-205 should have lower levels of ZEB1.

**Figure 7:**
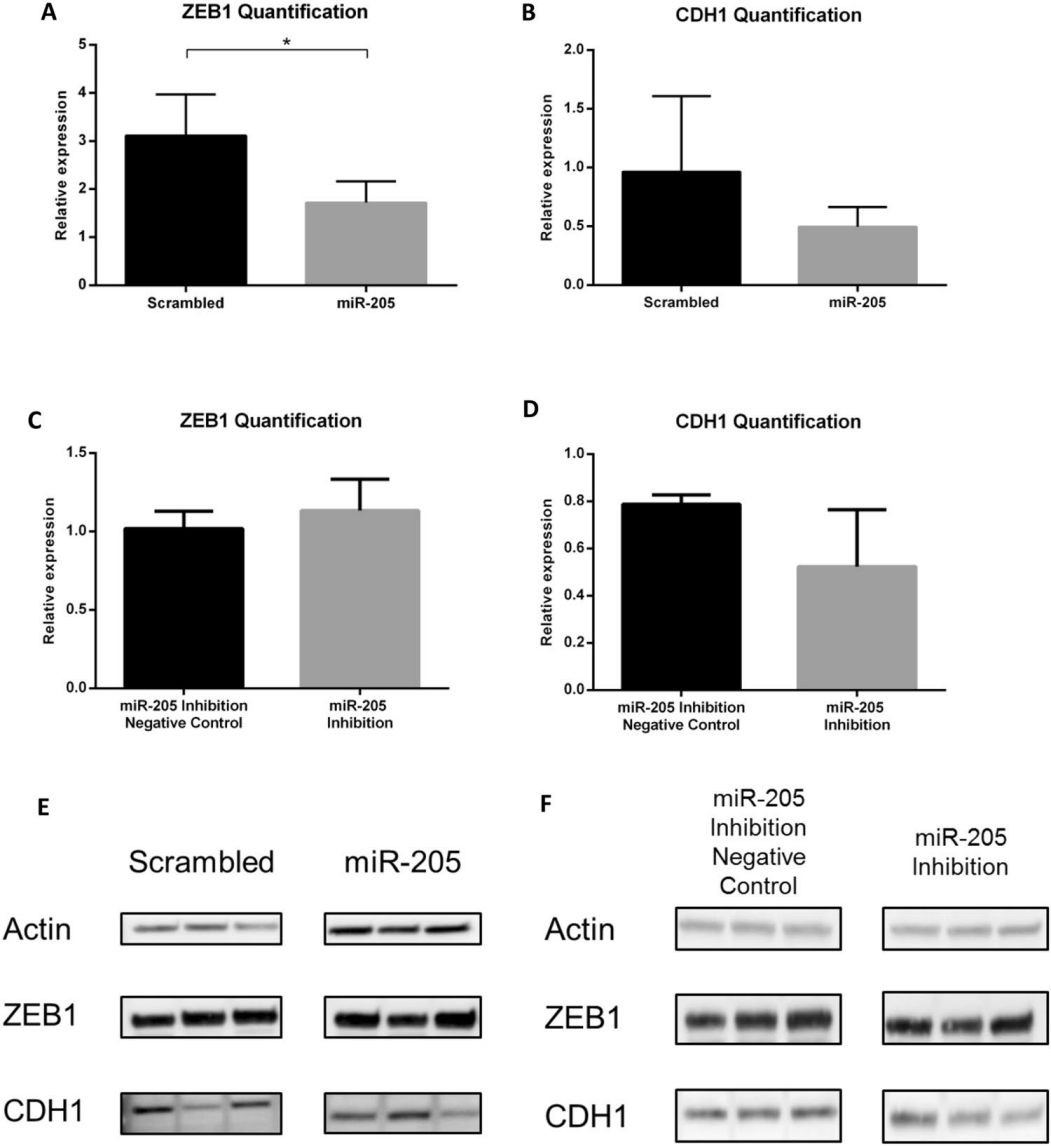
Functional validation of miR-205 overexpression and inhibition. (**A,B**) ZEB1 and CDH1 relative protein levels quantification by densitometry in ImageJ (normalized to actin) of miR-205 overexpressing cells and (**C,D**) of miR-205 inhibited cells. (**E**) ZEB1 and CDH1 bands intensities as assessed by western blot (Actin: 42KDa; ZEB1: ≈70 KDa; CDH1: ≈55KDa) in miR-205 overexpression and (**F**) in miR-205 inhibition condition. For comparison, scrambled and miR-205 overexpression samples were loaded on the same gel and miR-205 inhibition negative control and miR-205 inhibition samples on another gel.

As expected, ZEB1 protein was significantly reduced after miR-205 overexpression when compared to the scrambled condition, thus demonstrating its efficient inhibition by miR-205 (Fig. 7A,B and Supplementary Figures 1–3).

When miR-205 overexpression was reverted by inhibition, ZEB1 protein was again increased when compared to the negative control. Although the difference was not statistically significant, the expected tendency was clearly observed (Fig. 7C,D and Supplementary Figures 4–6). These results support the notion that miR-205 binds to ZEB1 gene and negatively regulates its expression at the protein level.

### Cell proliferation assays

**Figure 8:**
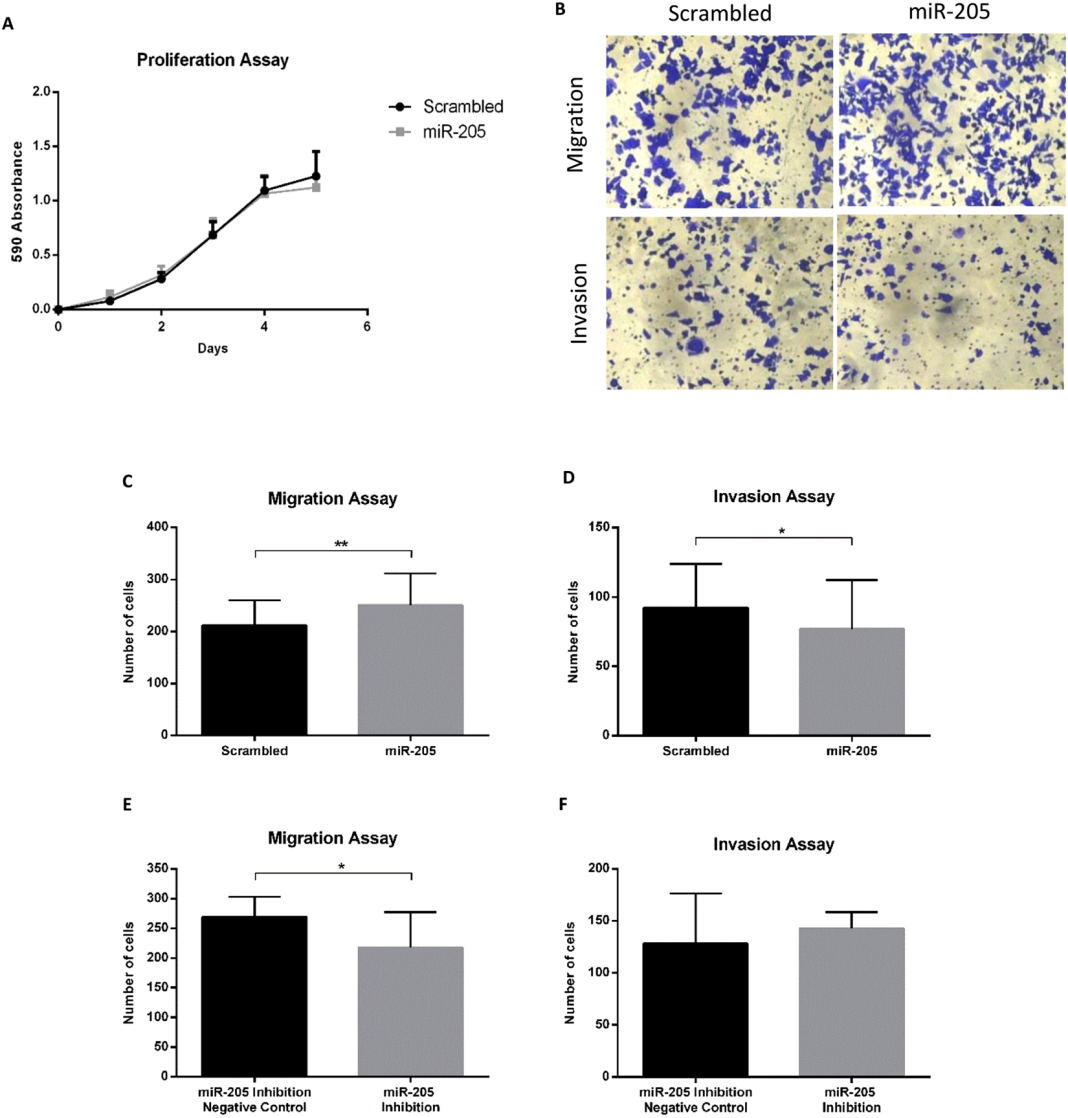
miR-205 overexpression and miR-205 inhibition functional effects on human melanoma cells. (**A**) No effect on proliferation was observed between miR-205 overexpressing A375 cells versus scrambled/control cells. Data represent the mean ± SD of 4 independent experiments. (**B**) A representative microscopic image of crystal violet staining of the migration and invasion assays in miR-205 overexpressing A375 cells and control cells. (**C** and **D**) Transwell migration (n = 3) and matrigel invasion (n = 3) assays on A375 cells. A375 cells transduced with miR-205 had higher migratory but lower invasive potentials than the control cells. (**E** and **F**) Transwell migration (n = 3) and matrigel invasion (n = 3) assays on A375 cells transfected with miR-205 inhibition negative control and miR-205-5p inhibitor. miR-205 inhibited cells migrated less but had higher invasive ability. Data represent the mean ± SD of 3 independent experiments. **p < 0.01, *p < 0.05.

Although low levels of miR-205 are associated with metastatic progression, after measuring absorbance for 5 days there were no differences between proliferation of A375 cells with and without miR-205 expression (Fig. 8A).

### Migration and Invasion Assays

miR-205 was overexpressed and then inhibited in A375 cells and migration and invasion assays performed for both conditions. After 48 hours, there were more miR-205 overexpressing cells that had migrated to the Transwell insert lower chamber in comparison with cells without miR-205 overexpression (p < 0.01). However, in the invasion assay, cells that expressed miR-205 invaded less than cells with no expression of the miR-205 (p < 0.05) (Fig. 8B–D).

As expected, after inhibition of miR-205 overexpression, opposite results were observed. Inhibited cells migrated less than miR-205 overexpressing cells (p = 0.011) whereas in the invasion assay cells with inhibited miR-205 overexpression invaded more although values did not reach significance (p = 0.325) (Fig. 8E,F).

## Discussion

Defining an accurate prognosis for patients with primary melanoma is an essential clinical goal. Presently, patient selection is based exclusively on the histopathological features. However, it is well known that patients with clinicopathologically analogous primary tumors can have different clinical outcomes. In addition to histopathologic characteristics, new molecular biomarkers are needed to better understand, diagnose, and treat melanoma patients. Moreover, only patients with advanced disease (distant metastasis and or unresectable regional metastases) currently benefit from immunotherapy and inhibitory molecules treatment, although their administration in high-risk patients before progression is under consideration.

It is widely accepted that miRNAs are dynamically involved in early and late events driving melanoma progression. Numerous studies highlight their potential as diagnostic and prognostic markers, as well as therapeutic targets in melanoma^10–18^. In this regard, van Kempen *et al.*^19^ found 11 miRNAs whose expression was associated with tumor thickness, and that loss of miR-200a, miR-200c and miR-203 expression was present at the invasive front in primary tumors. In particular, four miRNAs (miR-200b, miR-200c, miR-203, and miR-205) correlated with thickness; two of which (miR-200c and miR-205) maintained the same direction as in our data.

We hypothesize that alterations in the expression of certain miRNAs in the tumor microenvironment of primary melanomas may predict distant metastasis. We evaluated the prognostic value of the expression of ten miRNAs, which have already been implicated in melanoma cell migration and/or invasion, in primary cutaneous melanoma tissue, particularly for its ability to predict distant metastasis and, consequently, melanoma specific survival. For this purpose, we correlated miRNA expression with the clinicopathological prognostic factors and, more importantly, with the clinical outcome of the patients.

miR-21, miR-125b, miR-150, miR-155, miR-205 and miR-211 are dysregulated in melanoma^10^. We found that miR-125b, miR-182, miR-200c and miR-205 expression was higher in primary tumors than in melanoma metastases. In fact, it has been suggested that miR-200c and miR-205 downregulation in tumor cells is an essential early step in the development of metastasis because of its important role as promotor of cancer progression^20^ at least in part through the regulation of E-cadherin expression during the epithelial-mesenchymal transition targeting its transcriptional repressors ZEB1 and ZEB2^20^. miR-205 has been proposed as a tumor suppressor microRNA in melanoma^21^. Dar *et al*. reported that miR-205 mediates its effects on melanoma cells partially via suppression of the E2F family of transcription factors E2F1 which play a role in the control of cell cycle and the AKT pathway in melanoma cell lines^22^.

In our series, downregulation of miR-125b, miR-182, miR-200c and miR-205 occurs sequentially over the full spectrum of melanoma progression. Moreover, we found a lower intratumoral expression of these four miRNAs in primary melanomas that further disseminated to distant sites compared with those that did not metastasize. Similarly, by comparing primary tumors that metastasized to regional lymph nodes with those that did not, Glud and coworkers^23^ identified nine miRNAs as differentially expressed, and miR-125b was downregulated in those with positive lymph nodes. According to these authors, the overexpression of miR-125b, a regulator of melanogenesis and differentiation of melanocytes^24^, was associated with senescence in melanoma cells, and decreasing levels of apoptosis when miR-125b was inhibited in the primary cell lines^25,26^. Based on these studies, Kappelmann *et al.*^27^ reported that miR-125b controlled melanoma progression by directly targeting c-Jun, as well as the downregulation of miR-125b expression in melanoma cell lines. MLK3 (mixed lineage kinase 3) proliferation and invasion promoting proteins, are also miR-125b targets in melanoma^28^.

A reduction in proliferation and migration has been reported when the expression of miR-125b was restored in the cell lines, suggesting that miR-125b expression had a tumor suppressor function in melanoma^27^. Moreover, a recent study showed a decreased expression of miR-125b in primary tumors with positive sentinel lymph nodes, and concluded that miR-125b exerted a negative metastatic regulation on melanoma cell invasion *in vitro* and *in vivo* by targeting ITGA9, and therefore inhibiting the epithelial-mesenchymal transition in melanoma^29^.

Our *in vitro* results in melanoma cells with and without miR-205 functional expression demonstrate that the loss of miR-205 potentiates the matrigel invasive ability of melanoma cells, although interestingly, neither proliferation nor migration was increased. Since invasion assays best reproduce the *in vivo* environment, in which cancer cells must interact constantly with the extracellular matrix, our findings reveal that tumor cell interaction with the extracellular matrix is critical to miR-205 inhibition of metastatic dissemination. In this regard Liu *et al*. found that miR-205 downregulation did not modify proliferation of melanoma cells but increased both invasion and migration^30^. In contrast, other authors have found that miR-205 either suppresses melanoma cell proliferation^22^ or makes no difference related to invasion^31^.

The transcriptomic subtypes of TCGA classification of cutaneous melanoma showed a distinctive miRNA expression profile^32^. However, among our selected miRNAs, only miR-125b was found to be downregulated in the worse prognostic “keratin expressor” subtype of this classification. On the other hand, upper levels of miR-125b were found in the “MITF-low” subgroup associated with better survival. These results support our findings, since miR-125b significantly correlated with worse DMFS and MSS in our cohort. miR-182, miR-200c and miR-205 expression levels did not correlate with any TCGA transcriptomic subtypes. In an in silico analysis using the TGCA database, Lohcharienkal *et al.*^33^ also found a decreased expression of miR-200c and miR-182 in primary vs metastatic melanomas. These findings agree with our observations, since miR-200c and miR-182 expression were significantly decreased in the different stages of melanoma progression. However, the above study did not evaluate the clinical implications of these differences.

In conclusion, downregulation of tumor suppressive miR-125b, miR-200c and miR-205 in primary melanomas is an intrinsic property of primary tumors that might contribute to distant metastasis, and therefore to reduced survival. One of the mechanisms by which miR-205 influences melanoma metastasis and survival seems to be related to the interaction of melanoma cells with the extracellular matrix.

Overall, these findings reveal a key role for these three miRNAs in melanoma metastasis. Moreover, their intratumoral expression in primary tumors may serve as effective prognostic molecular biomarkers in order to select high-risk melanoma patients. Furthermore, potentiating the expression levels of these three miRNAs, and particularly that of miR-205, might have a potential therapeutic value in melanoma patients at the time of diagnosis of the primary tumor, by preventing metastatic dissemination and improving survival in the context of personalized medicine.

## Methods

### Human melanoma tissues

132 cryopreserved tumor specimens from patients with cutaneous malignant melanoma were selected for this study. Samples comprised primary melanomas (n = 65) and melanoma metastases (n = 67), of which 28 were in-transit, 24 regional lymph node and 15 distant metastases (4 skin, 3 lung, 2 brain, 2 soft tissue, 2 subcutaneous tissue, 1 bone, 1 liver). All tumor specimens were collected at the time of surgery at the Department of Anatomic Pathology, Hospital Clínico Universitario, Valencia, Spain, from November 2002 to December 2014.

Primary tumor parameters relevant to this study included Breslow thickness, mitotic index, ulceration, growth phase, location, gender, stage and histological type. Clinical follow-up, with particular emphasis on the development of distant metastases and melanoma mortality, ranged from 6.5 to 156 months (mean 71, median 68 months). All tumors were classified according to the 2017 American Joint Committee on Cancer (AJCC) staging system.

Both primary and metastatic tumor specimens were manually macrodissected to ensure maximum tumor tissue content. For primary melanomas, a tumor slice immediately adjacent to the thickest area of the tumor was selected for RNA extraction, and was immediately frozen in liquid nitrogen and stored at -80 °C. The remaining fresh tumor tissue from each case was formalin-fixed and paraffin-embedded for routine diagnosis. This protocol was approved by the Ethical and Scientific Committees, Hospital Clínico Universitario, Valencia, and all their guidelines were followed. Medical records from all patients were reviewed and clinical follow-up locked in December 2016. All patients provided written informed consent.

### Human melanoma and melanocyte cell culture

The following well-characterized melanoma cell lines have been used: A375, SKMEL-147 and 451 Lu. The latter was kindly provided by Dr. Eva Hernando (New York University Langone Medical Center, NY), SK-MEL-147 by Dr. Marisol Soengas (CNIO Melanoma Group) and human epidermal melanocytes by Dr. Julián Carretero (Universitat de València).

Human melanocytes were cultured in 254CF medium supplemented with calcium chloride at a final concentration of 0.2 mM, with Human Melanocyte Growth Supplement (Cascade Biologics Inc., Gibco), penicillin and streptomycin. Medium was changed daily.

Melanoma cells were grown in Dulbecco’s Modification of Eagle’s Medium (DMEM) with glucose and L-glutamine supplemented with 10% FBS, penicillin and streptomycin (Gibco) and incubated in a humidified incubator at 37 °C and 5% CO_2_. To avoid cultures above 80% confluency, subcultures were made regularly with 0.25% trypsin–EDTA (Gibco). All cells were routinely tested for Mycoplasma contamination using MycoAlert™ mycoplasma detection kit (Lonza).

### RNA extraction

mirVana miRNA Isolation Kit (Ambion) was used to perform total RNA extraction from patient melanoma tissue and cultured cells following the manufacturer’s recommendations. The procedure for tissues differed from that of cultured cells only in the cell lysis step.

Melanoma tissues were immediately disaggregated with a pre-chilled scalpel into tiny portions and were mechanically homogenized in 600 µl of Lysis/Binding buffer using the TissueLyser LT system (Qiagen). For cultured cells, 600 µl of Lysis/Binding solution were added and samples vortexed. All the following procedures were carried out in the same manner and according to the manufacturer’s protocol with minor modifications. RNA was quantified using Nanodrop ND-1000 Spectrophotometer (Thermo Scientific) and stored at -80 °C.

### miRNA quantification

From human samples and from cultured cells, relative quantification of mature microRNAs was carried out by reverse transcription quantitative Real time PCR (RT-qPCR). Reverse transcription (RT) was performed using TaqMan MicroRNA Reverse Transcription Kit using RNase inhibitor (Lifetechnologies). In 45-µl reactions, 200 ng of total RNA was converted to cDNA. RT reactions were multiplexed by customizing RT primer pool with miRNA-specific RT primers of interest following manufacturer’s recommendations^34,35^. The RT primers pooled were the provided along with the TaqMan MicroRNA Assays (Applied Biosystems) that are listed in Table 6.

**Table 6:** List of TaqMan ®MicroRNA assays used in this study.

Assay Name	Assay ID	Target sequence
hsa-miR-9–5p	000583	UCUUUGGUUAUCUAGCUGUAUGA
hsa-miR-21-5p	000397	UAGCUUAUCAGACUGAUGUUGA
hsa-miR-125b-5p	000449	UCCCUGAGACCCUAACUUGUGA
hsa-miR-137	001129	UUAUUGCUUAAGAAUACGCGUAG
hsa-miR-182-5p	002334	UUUGGCAAUGGUAGAACUCACACU
hsa-miR-191-5p	002299	CAACGGAAUCCCAAAAGCAGCUG
hsa-miR-200c-3p	002300	UAAUACUGCCGGGUAAUGAUGGA
hsa-miR-205-5p	000509	UCCUUCAUUCCACCGGAGUCUG
hsa-miR-211-5p	000514	UUCCCUUUGUCAUCCUUCGCCU
hsa-miR-221-3p	000524	AGCUACAUUGUCUGCUGGGUUUC
RNU6B	001093	CGCAAGGAUGACACGCAAAUUCGUGAAGCGUUCCAUAUUUUU
RNU48	001006	GAUGACCCCAGGUAACUCUGAGUGUGUCG
		CUGAUGCCAUCACCGCAGCGCUCUGACC

In brief, 1 µl of cDNA was used in a 10-μl qPCR reaction by adding TaqMan Universal Master Mix II, no UNG and TaqMan MicroRNA Assays for target miRNAs (Lifetechnologies). Small nuclear RNU48 was used as endogenous control. All reactions were performed in triplicate in 384-well plates on a 7900 HT Fast Real- Time PCR system (Lifetechnologies). Both, RT and qPCR negative controls were included for each assay.

Samples that showed Ct median values for the endogenous reference out of the range (from 17 to 23) were classified as not appropriate for normalization and, consequently, excluded from further qPCR analysis. For the relative quantification of miRNA expression, the Ct method was used and the results were analyzed using Expression Suite software (Lifetechnologies).

### miRNA overexpression

Two different miRNA expression constructs: a lentiviral construct and a non-viral expression plasmid were tested for efficiency of miR-205 overexpression and the most efficient (the lentiviral construct) was selected for further experimentation.

Lentiviral constructs for miR-205 (pmiRH-205) and scrambled negative control (pmiRH-Scr) were purchased from System Biosciences. The construct also carries a fluorescent marker (GFP) to monitor positive cells for transfection and transduction. First, lentiviral particles were produced by transient transfection of HEK293T cells with lentiviral expression constructs using Lipofectamine 2000 in Opti-MEM (Invitrogen).Lentiviral supernatant was filtered using 0.45 µm filters at 36 hours post-transfection and stored at −80 °C.

A375 target cells were seeded at a density that produced approximately 70% confluence in 24 hours and incubated overnight preceding infection. Medium was replaced with filtered 1:4 diluted viral supernatant containing 4 µg/ml polybrene (Sigma), and cells were then incubated for 4 hours before removing the viral supernatant followed by replacement with fresh medium. Transduction efficiency was checked for GFP expression. miR-205 overexpression was validated by RT-qPCR as detailed in miRNA quantification section.

### miRNA transient inhibitor transfection

Downregulation of miR-205 activity was achieved using mirVana inhibitors. Optimal transfection conditions that resulted in maximum miRNA inhibitor-mediated activity with minimal cytotoxicity were determined and maintained across experiments and controls. mirVana miRNA Inhibitors (negative control 4464076 and hsa-miR-205-5p ID MH11015) were transfected in 6-well plates for a final concentration of 50 nM using Lipofectamine (Lifetechnologies). Maximal inhibitor activity was achieved at 24 hours post-transfection.

### Protein isolation

Cell lysates were prepared in RIPA buffer freshly supplemented with sodium orthovanadate and protease inhibitors cocktail (Sigma) on ice. After centrifugation for 30 minutes at 14000 rpm at 4 °C, the supernatant was collected and protein concentration was determined by Lowry method reading absorbance at 660 nm on a multilabel plate reader (VICTOR™ X3 Multilabel Plate Reader, PerkinElmer). Standard curves were generated with BSA. Proteins from inhibition experiments were isolated 24 hours post-transfection.

### Western blotting

Cell lysates 50 µg of protein per lane) were resolved in Tris/glycine SDS-PAGE gradient (4–20%) gels (Mini-PROTEAN TGX Stain-Free gels, Bio-Rad) and transferred to PVDF membranes by semi-dry blotting (Bio-Rad Trans-Blot Turbo system). Membranes were blocked for 1 h at room temperature with 5% milk in TTBS. Membranes were probed with primary antibodies against AREB 6/ZEB1 (Abcam ab181451), E-Cadherin (CDH1) (Abcam ab40772) and as loading control b-Actin-Peroxidase (Sigma A3854) overnight at 4 °C, followed by appropiate HRP-conjugated secondary antibodies (Cell Signaling Anti-mouse IgG HRP-linked Antibody and Peroxidase AffiniPure Goat Anti-Rabbit IgG) except for actin.

Immunolabeled proteins were detected by incubation with the ECL-based SuperSignal West Pico PLUS Chemiluminescent detection Substrate (Thermo Scientific) using the ImageQuant LAS 4000 (GE Healthcare Life Sciences) imaging system. Sample and control band densities were quantified and compared with ImageJ. Data was normalized to actin.

Four replicates of scrambled and miR-205 overexpression were loaded on the same gel. For miR-205 inhibition negative control and miR-205 inhibition, three replicates per condition were loaded on the same gel. In all cases, all the replicates were used for each quantification in ImageJ. Three representative bands per condition were cropped from different parts of the same gel for the three genes per experiment. Full-length blots are included in Supplementary Figures 1–6.

### Proliferation assays

A375 cells were seeded at low density (1 × 10^3^ cells/well in 100 µl) in a 96-well plate. Before seeding, cell viability was analyzed by the trypan blue exclusion method. Cells were then fixed, washed and stained with Crystal Violet. Cells were destained with 15% acetic acid and absorbance was measured at 590 nm on a VICTOR™ X3 Multilabel Plate Reader (PerkinElmer). The absorbance value measured after 24 hours was used as a control and the relative absorbance value was calculated every 24 hours for 5 days. The experiment was repeated four times.

### Migration and invasion Assays

A 300 µl suspension of 4 × 10^4^ A375 transduced melanoma cells was added to Transwell inserts (Corning) uncoated or coated with Matrigel (Corning) diluted 1:40 in coating buffer for migration and invasion assays, respectively. Matrigel coated inserts were incubated for 2 hours at 37 °C. After coating, cells were seeded in the inner chamber in serum-free DMEM medium, and 500 µl of DMEM medium containing 10% FBS was added to the outer chambers. At counting for seeding, cell viability was analyzed by the trypan blue exclusion method. An equivalent volume of cell suspension was transferred to empty wells as cell input controls.

After forty-eight hours, cells were fixed (1% Glutaraldehyde in PBS), washed in PBS and stained with 0.5% Crystal Violet followed by extensive washing with H_2_O. Non-migrated or invasive tumor cells remaining on the top-side of the porous membrane were removed with a cotton swab. Cells that had migrated or invaded to the downside were photographed and scored by imaging and counting four fields per insert (inserts in triplicate per condition). All the experiments were repeated in triplicate. The migration and invasion assays were performed for transient transfection experiments with inhibitors as described above 24 hours post-transfection.

### Statistical analysis

Data plotting and statistical analysis were conducted using SPSS software package V.17.0 (SPSS Software, Inc.) and GraphPad Prism V.6.01 (GraphPad Software, Inc.).

Two-tailed Mann-Whitney test was used to analyze the association between miRNA expression and categorical clinicopathological parameters (two categories) while Kruskal-Wallis rank-sum test was used to compare categorical parameters defined by more than two different groups. The correlation between miRNA expression and continuous clinicopathological variables (Breslow thickness and mitotic index) was assessed by Spearman correlation.

Survival analysis was performed using Kaplan-Meier curves (Log-rank test). A Cox proportional hazard model was constructed using stepwise selection to identify independent predictors of clinical outcome, considering Hazard Ratios (HR), 95% CI and p values.

Distant metastasis free survival (DMFS) and melanoma specific survival (MSS) were defined as the period from the date of surgical excision of the primary melanoma to the date distant metastasis occurred or last follow-up (censored), or death from melanoma (event), respectively. For all statistical tests, a p value of less than 0.05 was considered statistically significant.

## Electronic supplementary material

Supplementary Information

## Data Availability

The authors will make materials, data and associated protocols promptly available to readers upon publication in Scientific Reports without undue qualifications in materials transfer agreements.
