# Supplementary material for: Downregulation of intratumoral expression of miR-205, miR-200c and miR-125b in primary human cutaneous melanomas predicts shorter survival
Source: Sci Rep. 2018 Nov 20;8:17076. doi: 10.1038/s41598-018-35317-3 (PMC6244285; doi:10.1038/s41598-018-35317-3)
Supplement: Supplementary file 1 — Supplementary Information [file 41598_2018_35317_MOESM1_ESM.docx]

**Downregulation of intratumoral expression of miR-205, miR-200c and miR-125b in primary human cutaneous melanomas predicts shorter survival.**

Authors:

Beatriz Sánchez-Sendra^1,2^, Carolina Martinez-Ciarpaglini^2^; José F González-Muñoz^2^, Amelia Murgui^3^, Liria Terrádez^4^, Carlos Monteagudo.^1,2,4,*^

Affiliations:

1. Department of Pathology, University of Valencia, Spain.
2. Biomedical Research Institute INCLIVA. Valencia, Spain.
3. Department of Biochemistry and Molecular Biology, University of Valencia, Spain.
4. Department of Pathology, Hospital Clínico Universitario de Valencia. Valencia, Spain.

**Supplementary Results**


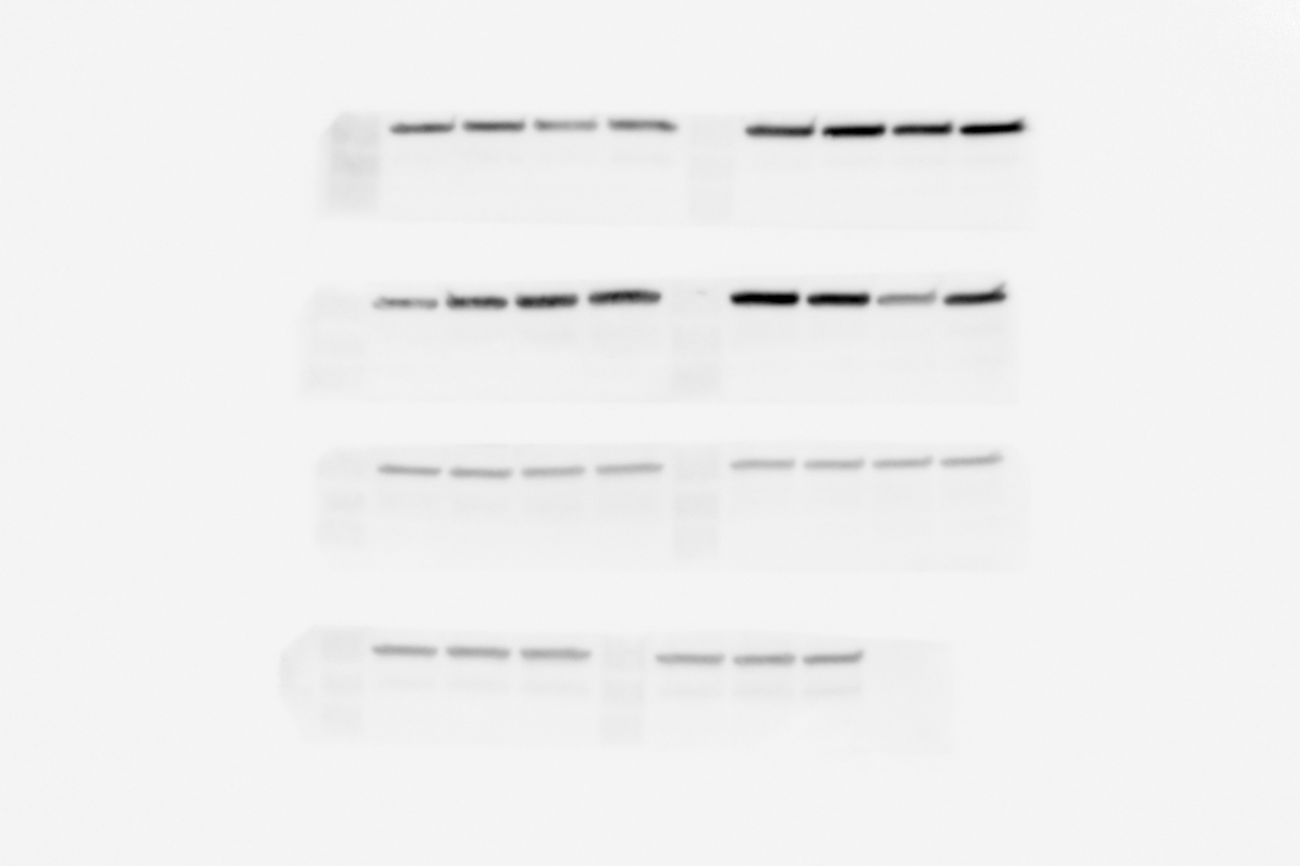


52kDa

42kDa

Scrambled

miR-205

Actin

**Supplementary Figure 1. Actin staining of the membrane for scrambled and miR-205 replicates for the western blot data in Figure 7 E**. The PVDF membranes were cut according to protein marker after transfer. Dashed lines indicate how images have been cropped for main figures. Exposure time: 8 seconds (standard mode).


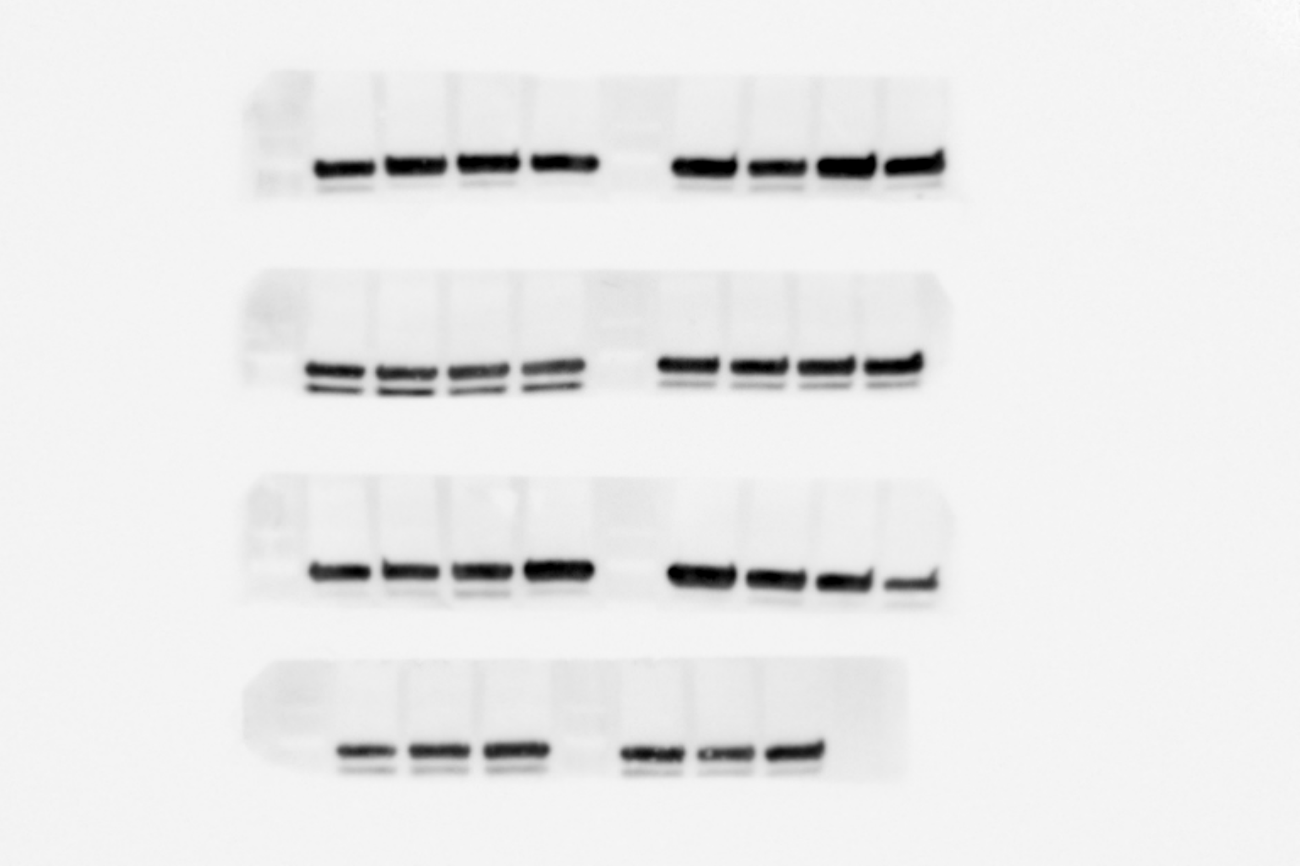


72kDa

52kDa

ZEB1

Scrambled

miR-205

**Supplementary Figure 2. ZEB1 staining of the membrane for scrambled and miR-205 replicates for the western blot data in Figure 7 E**. The PVDF membranes were cut according to protein marker after transfer. Dashed lines indicate how images have been cropped for main figures. Exposure time: 4 seconds (high mode).


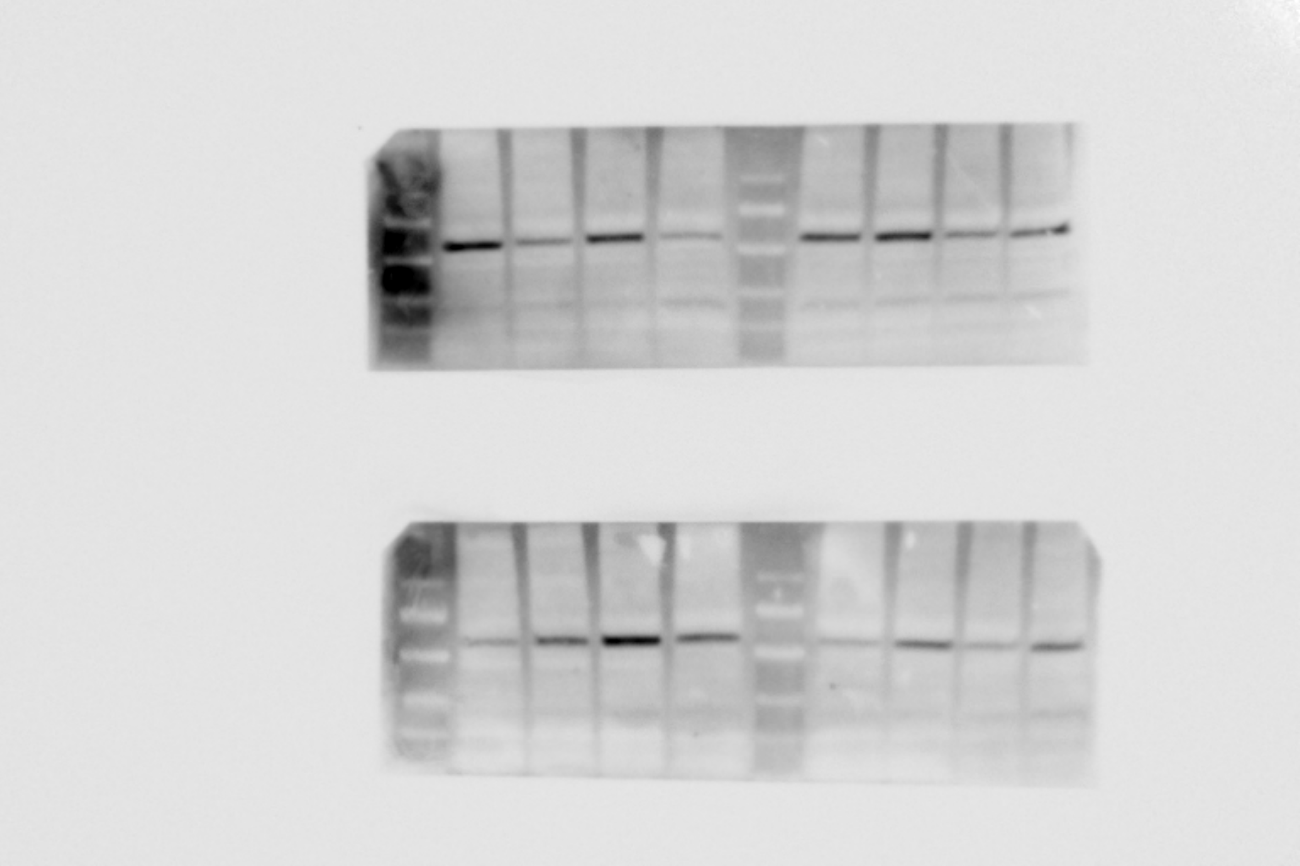


72kDa

52kDa

CDH1

Scrambled

miR-205

**Supplementary Figure 3. CDH1 staining of the membrane for scrambled and miR-205 replicates for the western blot data in Figure 7 E**. The PVDF membrane was cut according to protein marker after transfer. Dashed lines indicate how images have been cropped for main figures. Exposure time: 5 seconds (high mode).

miR-205
Inhibition

miR-205
Inhibition
Negative Control

52kDa

42kDa

Actin


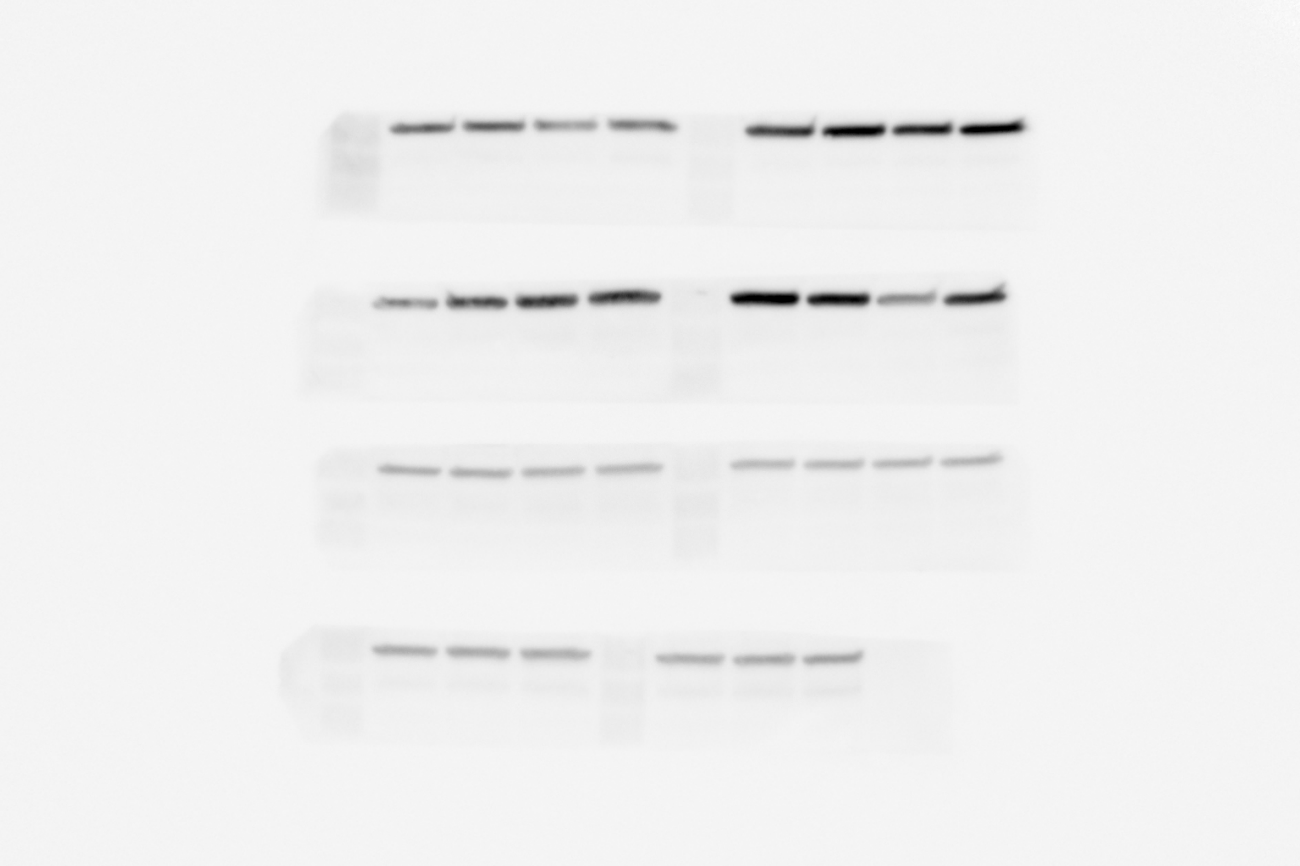


**Supplementary Figure 4. Actin staining of the membrane for pmiRH-205 inhibition negative control and pmiRH-205 inhibition replicates for the western blot data in Figure 7 F**. The PVDF membranes were cut according to protein marker after transfer. Dashed lines indicate how images have been cropped for main figures. Exposure time: 8 seconds (standard mode).

miR-205
Inhibition
Negative Control

miR-205
Inhibition


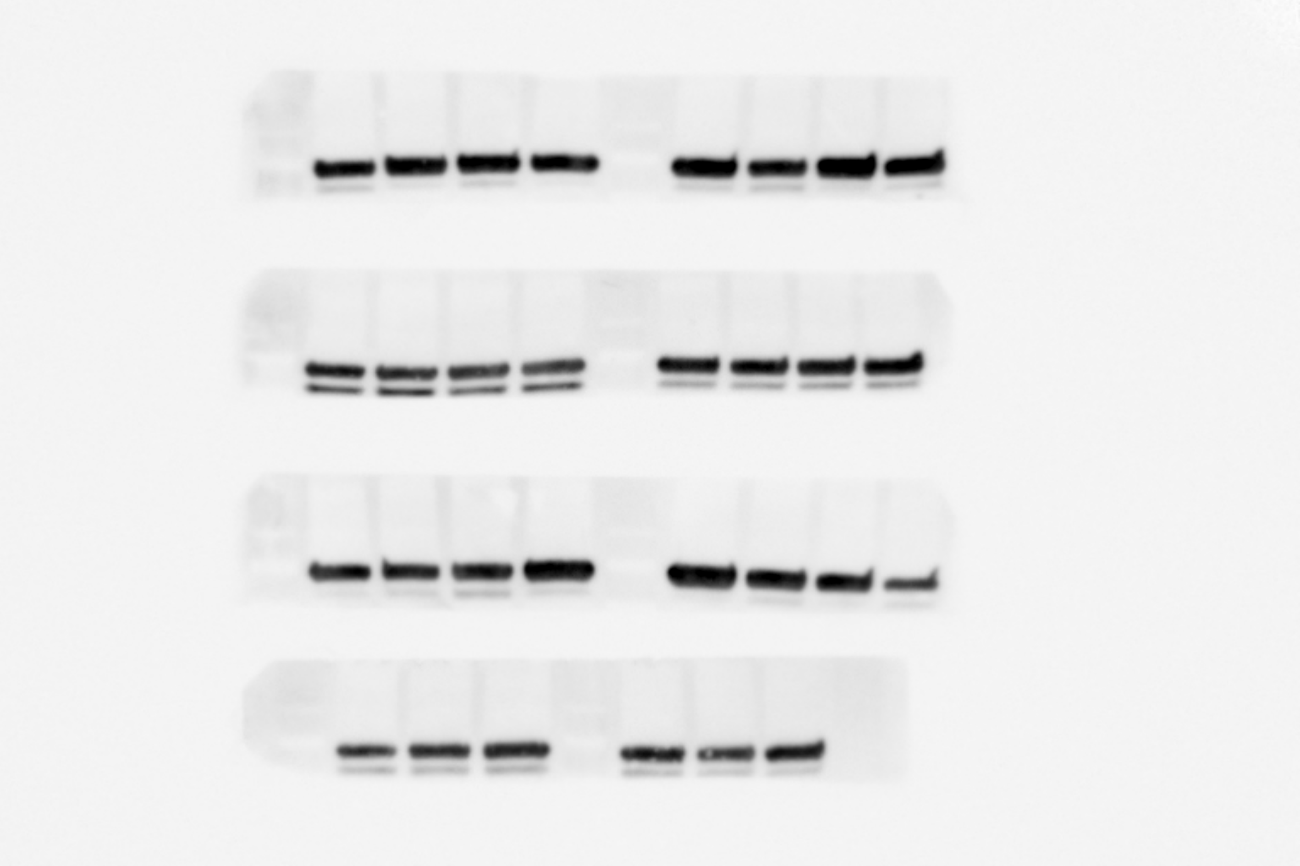


72kDa

52kDa

ZEB1

**Supplementary Figure 5. ZEB1 staining of the membrane for pmiRH-205 inhibition negative control and pmiRH-205 inhibition replicates for the western blot data in Figure 7 F**. The PVDF membranes were cut according to protein marker after transfer. Dashed lines indicate how images have been cropped for main figures. Exposure time: 4 seconds (high mode).

miR-205
Inhibition
Negative Control

miR-205
Inhibition


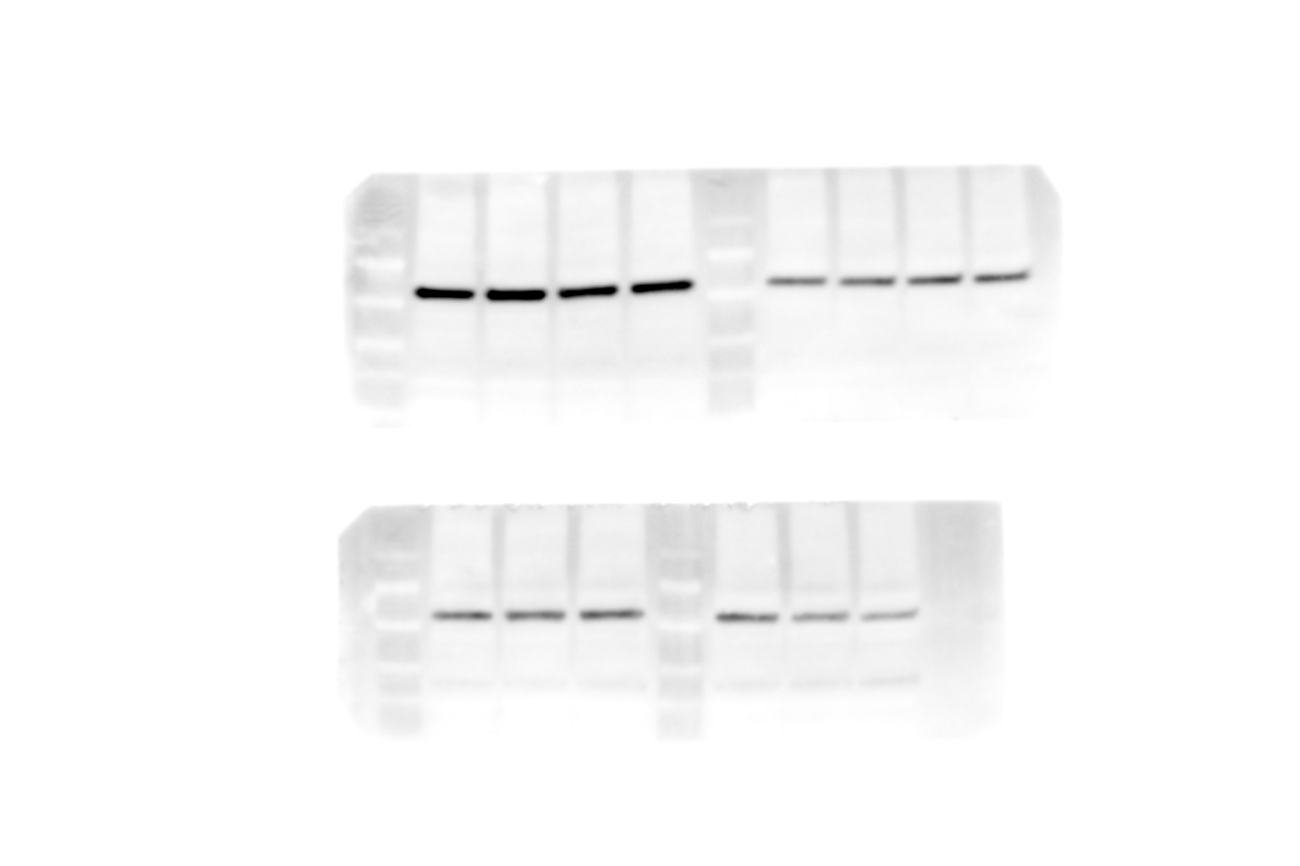


72kDa

52kDa

CDH1

**Supplementary Figure 6. CDH1 membrane staining of the membrane for pmiRH-205 inhibition negative control and pmiRH-205 inhibition replicates for the western blot data in Figure 7 F**. The PVDF membrane was cut according to protein marker after transfer. Dashed lines indicate how images have been cropped for main figures. Exposure time: 4 seconds (high mode).
